# Direct RNA Sequencing of Dengue and Chikungunya Viruses with Ultra-Low Input and Custom Multiplexing

**DOI:** 10.7171/001c.158995

**Published:** 2026-06-02

**Authors:** Clément Tanvet, Chloé Baum, Olivia O’Connor, Chloé Bohers, Myrielle Dupont-Rouzeyrol, Marc Monot

**Affiliations:** 1 Plate-Forme Technologique Biomics Université Paris Cité; 2 Dengue and Arboviruses Research and Expertise Unit Institut Pasteur de Nouvelle-Calédonie; 3 Arboviruses and Insect Vectors Université Paris Cité

**Keywords:** Direct-RNA sequencing, Nanopore, Dengue virus, Low RNA input, Multiplexing, chikungunya virus

## Abstract

Accurate viral genome characterization is essential for understanding viral dynamics. Oxford Nanopore Technologies (ONT) Direct RNA Sequencing provides detailed genomic data by directly reading RNA molecules, avoiding biases from reverse transcription and PCR. However, it requires high RNA inputs and lacks a multiplexing protocol, raising costs. This study explores Direct RNA Sequencing with minimal RNA inputs and multiplexing for arboviruses. We sequenced five strains, including dengue and chikungunya viruses, to test ultra-low RNA inputs and multiplexing. Results show successful sequencing and over 99% genome coverage with ultra-low inputs, highlighting the potential of Direct RNA Sequencing in genomic surveillance and the detection of RNA modifications. This method also shows promise for future application in sequencing patient serum samples without the need for viral cultivation.

## Introduction

In the field of virology, rapid and accurate characterization of viral genomes is essential for understanding their biology, pathogenic potential, and evolutionary dynamics through genomic surveillance. Achieving a near full-length genome sequencing of arboviruses at the single molecule level can be challenging.^1^ Oxford Nanopore Technologies (ONT) direct RNA sequencing offers an innovative approach for obtaining comprehensive genomic information by directly reading RNA molecules in their native form.^2^ This eliminates the need for a reverse transcription and prior amplification by polymerase chain reaction (PCR), thereby reducing sequencing biases and preserving native RNA features.

Although direct RNA sequencing has been successfully applied to viral genome sequencing,^3,4^ it presents certain limitations. It requires high amounts of RNA inputs (300 ng of poly(A)-tailed RNA or 1 µg of total RNA), quantities that are often difficult to obtain for viral samples. Additionally, the absence of a multiplexing protocol necessitates the use of a dedicated RNA flow cell for each sample, which substantially increases the cost per sample.

In this study, we evaluated the feasibility of sequencing arbovirus genomic RNA using very low input material and the feasibility of implementing a multiplexing strategy by sequencing several strains of arboviruses on the same flow cell. Using direct RNA, we sequenced five arbovirus strains, including three strains of dengue virus (DENV) and two strains of chikungunya virus (CHIKV) across two separate experiments. The first experiment (A) focused on the impact of decreasing input RNA quantities (50, 5, and 0.5 ng, respectively) for one strain of each arbovirus. The second experiment (B) evaluated the multiplexing of five arboviruses strains on the same flow cell. Prior to multiplexing, we first ensured that the percentage of genomic differences among the selected arboviruses strains was sufficient to enable unambiguous reading assignments based on mapping. Our results demonstrate that direct RNA sequencing is feasible even with ultra-low viral RNA input amount, achieving excellent coverage of nearly the entire genome. This approach also enabled the successful sequencing of a custom multiplexed library of five different arboviruses strains, resulting in read depth exceeding 60x and covering more than 99% of the reference genome for both native and poly(A)-tailed RNA genomes. These findings underscore the potential of direct RNA sequencing and hint at future possibilities for directly utilizing patient serum samples, thereby bypassing the requirement for viral cultivation.

## Materials and Methods

### Viral RNA Origins

The viral RNA samples used in this study were derived from five arbovirus strains isolated from the supernatant of C6/36 cells (*Aedes albopictus*) or VERO cells (mammalian cells) inoculated with patient serum; these samples originate from distinct geographical locations. These five strains include two CHIKV and three dengue viruses (DENV). One CHIKV was isolated from La Réunion Island in 2005 and belongs to the East-Central-South-Africa (ECSA) genotype (CHIKV_LR)^5^ while the second one was isolated from New Caledonia in 2011 and belongs to the Asian genotype (CHIKV_NC).^6^ The DENV include one serotype 1 isolated in New Caledonia in 2017, which belong to the genotype I (DENV-1_NC), and two serotype 2, which were isolated in New Caledonia in 2022 (DENV-2_NC) and in Thailand in 1974 (DENV-2_BGK)^7^ and belong to the Cosmopolitan and the Asian-I genotypes, respectively. The GenBank ID references for all five strains are reported in Table 1.

**Table 1.** Summary of sequencing and bioinformatics results for the two experiments (varying inputs and multiplexing)

	**Experiment A (varying inputs)**						**Experiment B (multiplexing)**				
Sample ID	CHIKV_LR			DENV-2_NC			CHIKV_LR	CHIKV_NC	DENV-2_NC	DENV-1_NC	DENV-2_BGK
RNA input (ng)	50	5	0.5	50	5	0.5	50	NQ	50	NQ	NQ
Library input loading (ng)	6	NQ	NQ	30	5	4	32				
Total reads	1,536,618	197.450	23,932	56,894	28,445	8,260	8,867,213				
Virus mapping reads (primary)	916,349 (56.6%)	115,839 (58.7%)	7,216 (30.1%)	58 (0.1%)	36 (0.1%)	0 (0%)	1,346,728 (15.2%)	26,008 (0.3%)	6,590 (0.1%)	33,457 (0.4%)	281,569 (3.2%)
Depth	282,020x	28,205x	1,688x	8x	7x	0x	417,441x	1,648x	804x	4,649x	42,370x
Reference genome ID (GenBank)	AM258992.1			OR136164.1			AM258992.1	HE806461.1	OR136164.1	MW315195.1	HE806461.1
Reads covering >99% of the viral genome	49,230 (5.4%)	3,000 (2.6%)	172 (2.4%)	2 (3.4%)	1 (2.8%)	0 (0%)	44,523 (3.3%)	67 (0.3%)	61 (0.9%)	84 (0.2%)	205 (0.1%)

Note: NQ = Nonquantifiable; RNA input = purified RNA and poly(A)-tailed (for DENV).

Viral stocks of CHIKV_NC and DENV-2_NC were obtained after two and four successive passages on C6/36 cells, respectively. Both viruses were originally isolated on C6/36 cells. For DENV-1_NC, two successive passages on VERO cells were required to produce a viral stock after the initial isolation on VERO cells. Viral RNA from these three samples was extracted with the QIAamp Viral RNA mini kit (Qiagen, Hilden, Germany), according to the manufacturer’s recommendations. Double elution using 2 x 30 µL of Buffer AVE was applied to recover the viral RNA from each sample. All RNA samples were stored at -80 °C.

Viral stocks of CHIKV_LR were produced after three passages on C6/36 cells. Regarding DENV-2_BGK, after two passages in *Ae. albopictus*, two others in *Toxorhynchites amboinensis*, and one in *Ae. aegypti* by intrathoracic inoculation, viral stocks were obtained by inoculating C6/36 cells. Both of their RNA extractions were performed following the Nucleospin® RNA Kit (Macherey-Nagel, Düren, Germany), RNA was individually eluted in 50 µL of RNase-free H₂O and stored at -80 °C.

### Direct RNA Sequencing Using Oxford Nanopore Technology

**Figure 1.**
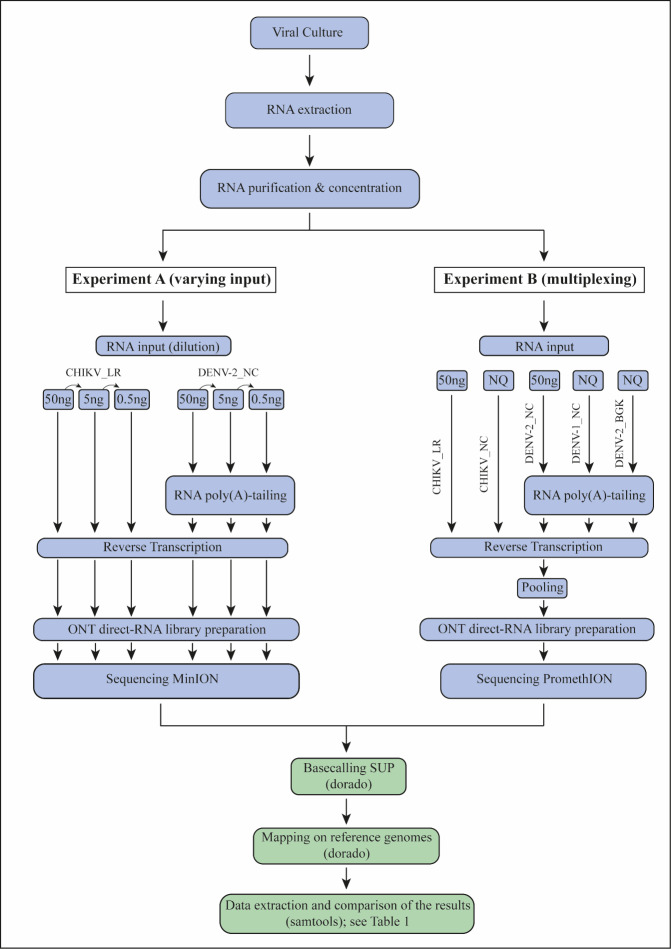
Summary of the experimental design including experiment A (varying input) and B (multiplexing).** Blue boxes represent steps performed up to sequencing. Green boxes represent bioinformatics and post-sequencing analyses. Note: NQ = Nonquantifiable.

The quantity of arboviral genomic RNA was measured using the Qubit 3.0 fluorometer (Thermo Fisher Scientific, Waltham, MA, USA) with the RNA high sensitivity kit and subsequently purified and concentrated using the RNA clean & concentrator kit (Zymo Research, Tustin, CA, USA). For DENV samples, a poly(A)-tail was added using *E. coli* poly(A) polymerase (New England Biolabs, Ipswich, MA, USA) following the ONT polyadenylation protocol. Libraries of CHIKV and poly(A)-tailed DENV samples were prepared using the direct RNA sequencing protocol from ONT (SQK-RNA004),^8^ including the reverse transcription optional step. Sequencing was performed on MinION RNA flow cells (FLO-MIN004RA) using a GridION for experiment A and on a PromethION RNA flow cell (FLO-PRO004RA) using a P2i for experiment B (Figure 1).

### Bioinformatics Process and Data Analysis

Raw POD5 files were processed for basecalling in SUP quality, demultiplexed, and trimmed of the ONT adapter sequence using Dorado/0.9.0 (https://github.com/nanoporetech/dorado). Sequencing quality control and analysis of the generated reads were conducted using qcnano version 0.5. Reads were aligned to the reference genomes using Dorado/0.9.0. Mapping data were extracted and compared between samples using Samtools/1.21.

## Results

In this study, we performed direct RNA sequencing on viral RNA from DENV and CHIKV to evaluate whether using very low RNA inputs (below the specifications recommended by ONT protocols) could still produce reads mapping to the reference genomes. We assessed whether these conditions could yield enough high-quality reads covering more than 99% of the complete viral genomes. Additionally, we aimed to evaluate a multiplexing strategy that allows sequencing of multiple arboviruses on the same flow cell, reducing the cost per sample given the absence of official multiplexing protocols for the nanopore direct RNA kit.

As previously described, these two objectives were tested in experiments A and B, respectively. The workflow for these experiments is presented in Figure 1.

The total number of reads obtained after sequencing the different samples at different RNA, as well as for the multiplexing experiment, is reported in Table 1. Mapping results for each sample are also provided in Table 1, showing that reads covering more than 99% of the full reference genome length were obtained. Although reads spanning the entire genome length were obtained, direct RNA nanopore sequencing is known to encounter challenges with 5’ end coverage of RNA molecules.^9^ Consequently, only reads covering over 99% of the reference genome were considered for comparative analysis.

Notably, the ultra-low RNA input (i.e., 0.5 ng) resulted in 172x coverage of the near full-length CHIKV_LR genome (>99%). In contrast, DENV-2_NC with 0.5 ng of RNA input did not demonstrate any reads mapping to the reference genome. This issue may be attributed to the additional and technically challenging poly(A)-tailing step required for DENV compared to CHIKV samples. However, the multiplexing experiment (B) yielded reads mapping to the reference genome for all five samples, including DENV for which the RNA input was not quantifiable.

Furthermore, experiment B showed robust sequencing performances, with read depths covering >99% of the full reference genome length for all five samples. Coverage depths ranged from 61x to 44,523x, despite some samples having nonquantifiable RNA inputs. Those depths could allow us to detect RNA modifications and methylation (e.g., m6A, m5C, inosine, and pseudouridine) as reported in recent studies.^10^

## Conclusion

Based on the results of our experiments, we report that direct RNA sequencing is feasible with ultra-low inputs of viral RNA (i.e., at concentrations up to 600 times lower than those recommended by ONT) while still achieving excellent read depth and near full-complete genome coverage (>99 %). In addition, our custom multiplexing library was successfully sequenced using direct RNA, resulting in read depths exceeding 60x and covering over 99% of the reference genome length. This was achieved for RNA genomes of five distinct arboviruses strains, including genomes bearing either native or enzymatically added poly(A) tails. Overall, these results highlight the potential use of direct RNA sequencing from patient serum without the need for viral cultivation for genomic surveillance and/or detection of RNA epigenetic modifications.

## Data Availability

Raw base-called sequences were deposited in the Sequence Read Archive under the BioProject Accession PRJNA1394482.
